# The Efficacy and Safety of PD-1/PD-L1 Inhibitors in Combination with Conventional Therapies for Advanced Solid Tumors: A Meta-Analysis

**DOI:** 10.1155/2020/5059079

**Published:** 2020-05-06

**Authors:** Run-Cong Nie, Chong-Bang Zhao, Xiao-Wei Xia, Ying-Shan Luo, Ting Wu, Zhi-Wei Zhou, Shu-Qiang Yuan, Yun Wang, Yuan-Fang Li

**Affiliations:** ^1^Department of Gastric Surgery, Sun Yat-sen University Cancer Center; State Key Laboratory of Oncology in South China; Collaborative Innovation Center for Cancer Medicine, Guangzhou, China; ^2^Department of Psychiatry, The Third Affiliated Hospital, Sun Yat-sen University, No. 600 Tianhe Road, Guangzhou, China; ^3^Department of Radiation Oncology, Sun Yat-sen University Cancer Center; State Key Laboratory of Oncology in South China; Collaborative Innovation Center for Cancer Medicine; Guangdong Key Laboratory of Nasopharyngeal Carcinoma Diagnosis and Therapy, Guangzhou, China; ^4^Department of Hematologic Oncology, Sun Yat-sen University Cancer Center; State Key Laboratory of Oncology in South China; Collaborative Innovation Center for Cancer Medicine, Guangzhou, China

## Abstract

**Objectives:**

To evaluate the efficacy of immuno-oncology combinational therapy (IOCT) versus monotherapy with programmed cell death 1 (PD-1) or PD-ligand 1 (PD-L1) inhibitors or conventional therapies, i.e., non-IOCT, in patients with advanced solid tumors.

**Methods:**

We systematically searched the PubMed, Embase, and Cochrane Library databases from January 2015 to October 2018 for eligible studies. We included randomized trials of IOCT with available hazard ratios (HR) for death. The random effects model was used to calculate pooled HR for death; heterogeneity was assessed using *I*^2^ statistics. The main outcome measure was overall survival (OS).

**Results:**

After screening 483 relevant articles, we identified twelve trials comprising 5388 patients for quantitative analysis. IOCT-treated patients had significantly higher tumor response rate (relative risk (RR): 2.51, 95% confidence interval (CI): 1.82-3.47), prolonged progression-free survival (HR 0.62, 95% CI: 0.53-0.74), and OS (HR 0.69, 95% CI: 0.61-0.78), compared with non-IOCT–treated patients. Sensitivity analyses also demonstrated the OS advantage of IOCT across different combination modalities, intervention agents, malignancy types, and PD-L1 expression (all *P* < 0.05). Notably, there were higher odds of high-grade (grade ≥ 3) adverse events with IOCT (RR: 1.81, 95% CI: 1.13-2.90), but the risk of treatment-related death (RR: 1.16, 95% CI: 0.84–1.60) was not increased compared with non-IOCT.

**Conclusions:**

IOCT is a preferable treatment option over PD-1/PD-L1 inhibitor monotherapy and conventional therapy for patients with advanced solid tumors. However, we should note the increased incidence rate of high-grade AEs in IOCT.

## 1. Introduction

Immune checkpoints are a series of coinhibitory and costimulatory receptors and ligands that control the process of immune suppression and evasion of malignant cancer cells, which are known as one of the hallmarks of cancer [1]. The programmed cell death 1 (PD-1) and programmed cell death ligand 1 (PD-L1) axis is one of the most important immune checkpoints as well as a valuable therapeutic target because it not only plays a key role in physiological immune homoeostasis, but also appears to be a means through which cancer cells evade the immune system [2]. The development and application of antibodies targeting PD-1 (nivolumab and pembrolizumab) and PD-L1 (atezolizumab, avelumab, and durvalumab) have advanced the treatment of melanoma [3], nonsmall cell lung cancer (NSCLC) [4], renal cell cancer [5], colorectal cancer [6], and head and neck cancer [7]. Currently, PD-1 or PD-L1 inhibitors are being investigated in more than 1000 clinical trials and are licensed to treat a variety of cancers by the U.S. Food and Drug Administration (FDA).

Nonetheless, although immuno-oncology therapy (IOT) is greatly advantageous in that it covers a wide range of tumor types, many shortcomings remain. Principally, the majority of patients could not achieve satisfactory treatment effects from immuno-oncology (IO) monotherapy due to the low overall response rate, varying from 20% to 40% [2, 8–13]. Using NSCLC as an example, IO monotherapy only improves the overall survival of a minority of patients that with PD-L1 expression ≥ 50% [11, 14]. Additionally, PD-1/PD-L1 inhibitors rely heavily on the tumor microenvironment to work; theoretically, only a fraction of patients with inflamed tumor could benefit from immunotherapy, and other immune types such as the immune-desert phenotype and immune-excluded tumors have poor response partly due to the absence of immune effector cells in the tumor microenvironment or obstruction between the immune effector cells and tumor cells [15]. Furthermore, IOT is associated with several immune-related adverse events [16] and requires an extremely high cost, as estimated as more than £234 000 (€258 000; $300 000) per quality adjusted life year [17]. Hence, much remains to be done before IOT can be extensively used in cancer treatment, and an immediate priority is improving the therapeutic efficacy of immunotherapy. To address these issues, substantial clinical trials are underway to explore whether combination with other therapies could improve the treatment effect of IOT.

To date, more than 1100 trials on several combinational modalities, such as IOT plus IOT (namely ipilimumab), chemotherapy, and targeted therapy, are underway for numerous cancer types [18]; initial inspiring results have been achieved with the combinations of IOT plus IOT [19] and IOT plus chemotherapy [20]. Nonetheless, as IOT clinical trials usually require long follow-up duration and large sample sizes to achieve statistical differences and have inconsistent results (both survival outcomes and adverse events [AEs]) among different trials [19–30], it is therefore essential to conduct a meta-analysis to pool the results of the available trials to explore the therapeutic efficacy and safety of IO combination treatment (IOCT) across different tumor types and between IOCT vs. PD-1/PD-L1 inhibitor monotherapy or conventional therapies (non-IOCT) to provide critical and useful information for the clinical utility of IOCT.

## 2. Methods

This study was conducted in compliance with Cochrane Handbook for Systematic Reviews of Interventions recommendations and was reported based on Preferred Reporting Items for Systematic Reviews and Meta-Analyses (PRISMA) statement guidelines [31].

### 2.1. Search Strategy and Selection Criteria

This is a trial-level meta-analysis. RCN and CBZ conducted a comprehensive systematic search of the Medline (PubMed), Embase, and Cochrane Library databases from January 2015 to October 2018 with no language restrictions to identify randomized controlled trials (RCT) of IOCT for advanced solid tumors. The main keywords were nivolumab, pembrolizumab, avelumab, atezolizumab, durvalumab, PD-1, PD-L1, checkpoint inhibitors, phase 2 trial, phase 3 trial, and randomized trial (see Supplementary Material (available here)). To be eligible, the RCT had to meet several prespecified criteria: population: enrolled patients with advanced solid tumors irrespective of site; experimental intervention: treated with PD-1 or PD-L1 inhibitors in combination with other treatment modalities irrespective of dosage and duration; control group: treated with anti-PD-1/PD-L1 single agent, ipilimumab, chemotherapy, or targeted therapy; and main outcome: reported outcome of overall survival measured as hazard ratios (HR). We excluded phase 1, nonrandomized phase 2 studies, retrospective, prospective observational cohort, reviews, basic science studies, quality of life studies, case reports, cost-effectiveness analyses, commentaries, conference abstracts without published full text original articles, and editorials. Furthermore, we examined the reference lists of all RCT fulfilling the eligibility criteria for any eligible studies missed by the initial search. Discrepancies in the literature search and inclusion were resolved by discussion and consensus.

### 2.2. Data Extraction and Risk of Bias Assessment

RCN, CBZ, and YSL extracted the reported HR for overall survival and the following clinicopathological characteristics of each eligible trial: article title, accrual period, phase of study, underlying malignancy, line of therapy, treatment regimen, patient number, PD-L1 expression, and median follow-up time. We used the Cochrane Risk of Bias Tool [32] to evaluate the risk of bias of every trial and scored it as high, low, or unclear risk of bias based on the following criteria: random sequence generation, allocation concealment, blinding, incomplete outcome data, and selective reporting.

### 2.3. Statistical Analysis

The primary endpoint of the present study was overall survival, defined as the time from randomization to death from any cause. The secondary endpoints were progression-free survival (the time from randomization to first RECIST 1.1- (response evaluation criteria in solid tumors-) defined progression or death), objective response rate (the percentage of patients with a confirmed best response of complete response or partial response according to RECIST 1.1), and treatment safety. AEs were graded according to the National Cancer Institute Common Terminology Criteria for Adverse Events, with grade 3, 4, or 5 considered severe. We calculated the treatment effects (HR or relative risk (RR)) of IOCT vs. non-IOCT, with 95% confidence interval (CI). Statistical heterogeneity between different trials and subgroups were assessed by the Cochrane Q statistic, and the extent of inconsistency contributing to the heterogeneity across different studies was assessed by *I*^2^ [33]. We considered *I*^2^ > 50% to indicate substantial heterogeneity. In the present study, the pooled HRs for death were calculated using the random effects model. Subgroup analyses were conducted based on combination modality, intervention agent, intervention agent target, type of control group, malignancy type, and PD-L1 expression. Sensitivity analyses were performed with restriction to large trials (>400 patients) and trials with mature follow-up time (median follow − up ≥ 24 months).

Potential publication bias was assessed via visual inspection of a funnel plot and evaluated using Begg's regression asymmetry tests [34]. All analyses were performed using Stata version 12.0 (StataCorp, College Station, TX, USA). All tests were two sided; *P* < 0.05 was considered statistically significant.

## 3. Results

### 3.1. Literature Search

**Figure 1:**
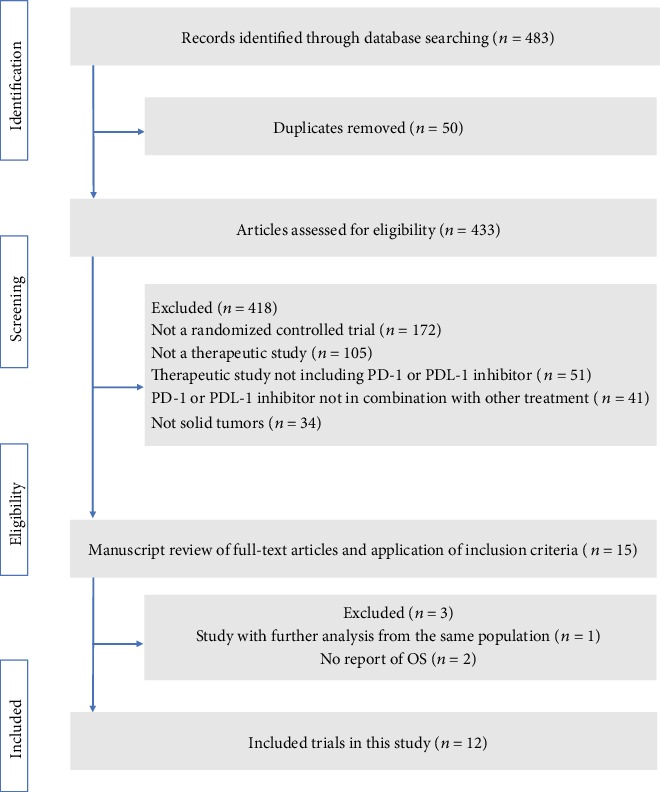
Flowchart diagram of study inclusion and exclusion. PD-1: programmed cell death 1; PD-L1: programmed cell death ligand 1; OS: overall survival.

After initial systematic literature review, we identified a total of 483 articles on the topic; 468 articles were excluded because they did not meet the inclusion criteria after eligibility screen of the titles and abstracts. We carefully reviewed the full texts of the remaining 15 potentially eligible papers and identified one duplicate report of the same data and two that did not report overall survival. The CheckMate 032 [21] was an open-label, phase 1/2 trial, and we included the two arms of nivolumab 3 mg/kg plus ipilimumab 1 mg/kg (54 patients) and nivolumab 3 mg/kg (98 patients). Hence, 12 RCT met the inclusion criteria for final analysis [19–30].  Figure 1 details the study selection process.

### 3.2. Study Characteristics

The 12 eligible RCT included in the present study involved 5388 patients: 2758 (51.2%) were treated with IOCT, and 2630 (48.8%) were treated with non-IOCT.  Table 1 details the characteristics of the RCT. The included studies were all international, multicenter RCT (5 phase 2 and 7 phase 3) funded by the pharmaceutical industry and published between 2016 and 2018 and provided fundamental evidence for the FDA to license these drugs. Most of the treatment line was first line therapy. The combinational modalities included IOT plus IOT (ipilimumab) (six trials), IOT plus chemotherapy (five trials), and IOT plus chemoradiotherapy (one trial). The CheckMate 067 trial [30] had one IOCT cohort (nivolumab plus ipilimumab group) and two non-IOCT cohorts (nivolumab group and ipilimumab group), leading to 13 comparisons in our analysis. The median follow-up varies from 7.8 months to 38 months. The method quality of the included trials was generally moderate to good; the main issue affecting quality was lack of blinding, where six trials were open-label (Supplementary Table 1).

### 3.3. Therapeutic Efficacy of IOCT for Overall Survival

**Figure 2:**
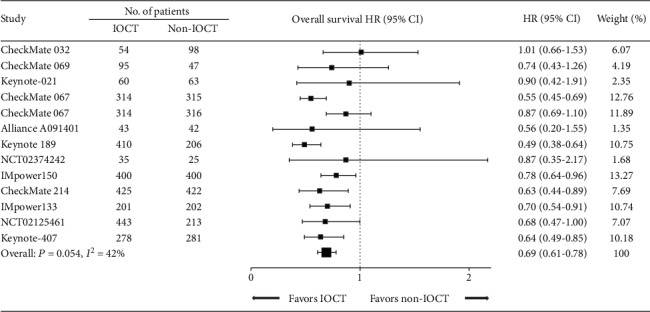
Forest plot of HRs comparing overall survival in patients who received IOCT vs. non-IOCT. Studies are listed on the left with respective number of patients of each treatment, HR with 95% CI, and weight are on the right. HR: hazard ratio; IOCT: immuno-oncology combination treatment; CI: confidence interval.

First, we calculated the treatment effect of IOCT vs. non-IOCT: IOCT-treated patients had significantly reduced risk of death in seven comparisons (HR: 0.49-0.78, all *P* < 0.05; Figure 2) of large sample sizes (>400 participants). Nonetheless, survival benefit was not reached in the other six comparisons (HR: 0.56-1.01, all *P* > 0.05; Figure 2), among which five had small sample sizes. Notably, moderate heterogeneity was observed in the overall treatment effect across the 13 comparisons (*P* = 0.054, *I*^2^ = 42.0%); therefore, the random effects model was preferred for the pooled analysis. Overall, IOCT was associated with significantly higher overall response rate (pooled RR: 2.51, 95% CI: 1.82-3.47, *P* < 0.001, Supplementary Figure S1A), prolonged progression-free survival (pooled HR: 0.62, 95% CI: 0.53-0.74, *P* < 0.001; Supplementary Figure S1B), and overall survival (pooled HR: 0.69, 95% CI: 0.61-0.78, *P* < 0.001; Figure 2), compared with non-IOCT.

### 3.4. Subgroup and Sensitivity Analyses

**Figure 3:**
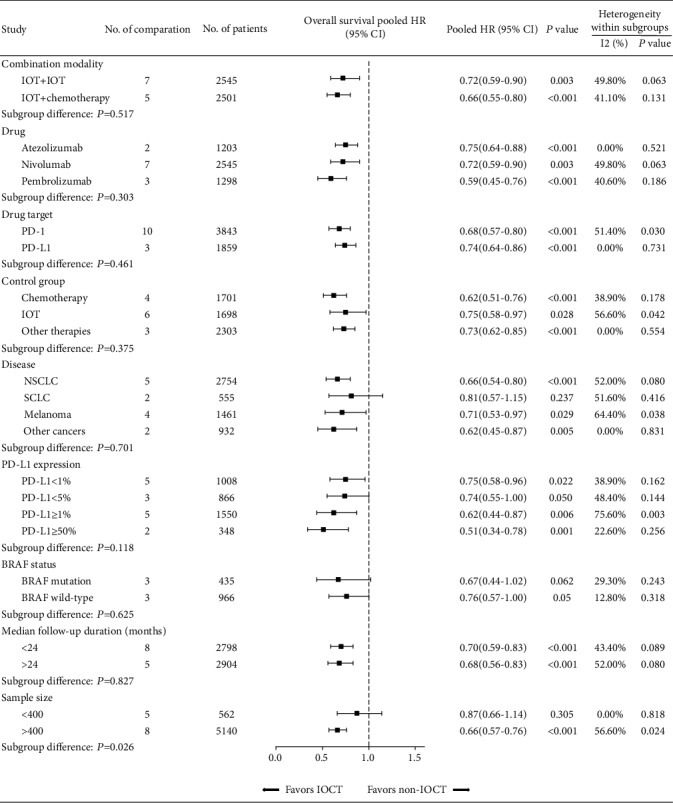
Subgroup and sensitivity analyses stratified by treatment, disease, and trial characteristics. HR: hazard ratio; IOCT: immuno-oncology combination treatment, CI: confidence interval.

To further evaluate the therapeutic efficacy of IOCT, we performed subgroup analyses according to the treatment, patient, and trial factors. Mainly, both the combinational modalities of IOT plus IOT (pooled HR: 0.72, 95% CI: 0.59-0.90, *P* = 0.003; Figure 3) and IOT plus chemotherapy (pooled HR: 0.66, 95% CI: 0.57-0.78, *P* < 0.001; Figure 3) showed overall survival benefit for patients. Furthermore, in subgroup analyses stratified by intervention agent, drug target, and control group identified consistent therapeutic efficacy among these subgroups (all pooled HR < 1.0; all *P* < 0.05). Additionally, in subgroups of different cancer types, there was significant survival benefit in patients with different malignancy types (pooled HR for NSCLC, melanoma, and other cancers: 0.66, 0.71, and 0.62, respectively, all *P* < 0.030; Figure 3), with the exception of SCLC (pooled HR: 0.81, 95% CI 0.57-1.15, *P* = 0.237, Figure 3), partly due to the limited number of patients involved (555 patients in two comparisons). These findings further indicate that IOCT can reduce risk of death regardless of combinational modality, interventional PD-1/PD-L1 inhibitor, and cancer type.

Next, we explored the IOCT treatment effect in subgroups according to PD-L1 expression. There was overlap among patients with different PD-L1 expression levels due to the multiple thresholds of PD-L1 expression reported in some trials. In summary, IOCT was effective in reducing risk of death in patients with high and low PD-L1 expression (pooled HRs for PD − L1 ≥ 50% (0.51), ≥1% (0.62), <5% (0.74), and < 1% (0.75), respectively, all *P* ≤ 0.050; Figure 3), indicating that IOCT was less reliant on pretreatment PD-L1 expression level when combined with other therapies. Interestingly, a tendency for reduction in pooled HR persisted with higher PD-L1 expression.

### 3.5. Safety of IOCT vs. Non-IOCT

**Figure 4:**
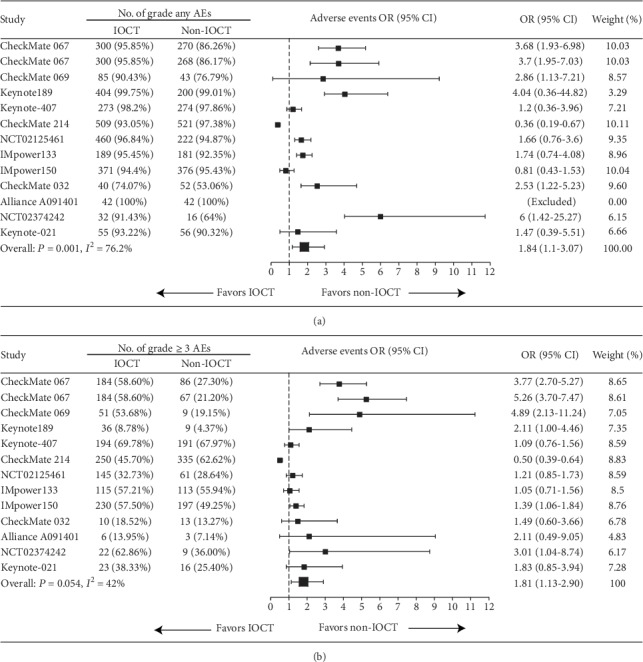
Forest plot of ORs comparing high-grade treatment-related AEs in patients who received IOCT vs. non-IOCT. Studies are listed on the left with respective number of patients of each treatment, OR with 95% CI and weight are on the right. (a) AEs regardless of the grade; (b) grade ≥ 3 AEs. OR: odds risk; IOCT: immuno-oncology combination treatment, CI: confidence interval; AE: adverse event.

The incidence of AEs of IOCT varies from 74.7% to 100%, compared to those of IOT of 53.1% to 100% (Figure 4(a)). Safety analyses of adverse events showed that IOCT had higher odds of any AEs (pooled RR: 1.84, 95% CI: 1.10-3.07, *P* = 0.020, Figure 4(a)).  Table 1 presents the incidences of high-grade (grade ≥ 3) AEs of each trial. IOCT had a tendency towards high-grade AEs in the majority of trials compared with non-IOCT; this was more obvious in trials of IOT plus IOT. Overall, IOCT-treated patients had higher odds of high-grade AEs (pooled RR: 1.81, 95% CI: 1.13-2.90, *P* = 0.012, Figure 4(b)) compared with non-IOCT–treated patients. Nonetheless, IOCT did not increase the risk of treatment-related death compared with non-IOCT (mortality rate, IOCT vs. non-IOCT: 0-8.2% vs. 0-6.6%; pooled RR: 1.16, 95% CI: 0.84–1.60, *P* = 0.375).

### 3.6. Publication Bias

We did not identify substantial asymmetry in visual inspection of the Begg's funnel plot (Supplementary Figure S2), which the Begg's rank correlation and Egger linear regression tests confirmed, indicating no evidence of publication bias (*P* = 0.542).

## 4. Discussion

IOCT represents a promising treatment modality for malignancies. Recently, several RCT have reported inspiring survival outcomes in patients who received IOT in combination with other therapies, such as ipilimumab [30], vascular endothelial growth factor (VEGF) inhibitor (bevacizumab) [25], and chemotherapy [28]. To the best of our knowledge, the present study is the first pooled analysis to summarize the therapeutic efficacy and safety of IOCT of these studies. Using the published data from 12 high-quality RCT that comprised 5388 patients with five different types of advanced solid tumors, our pooled analysis reveals that, compared with non-IOCT, IOCT was significantly associated with 38% reduction in the risk of progression and 31% reduction in the risk of death. The overall survival advantage of IOCT was identified across different combination modalities, intervention agents, and malignancy types. Interestingly, when combined with other therapies, IOT showed survival benefit for patients with high and low pretreatment PD-L1 expression (pooled HR: 0.51-0.75, all *P* ≤ 0.050), indicating that IOCT has antitumor activity without the restriction of pretreatment PD-L1 expression.

Contrary to conventional therapies, such as chemotherapy and targeted therapy, IOT relies heavily on the tumor microenvironment and antitumor immunity, both of which are dynamically altered during tumor microenvironment–immunity interaction [35–37]. Combining other therapies with IOT may potentially modify the tumor immune microenvironment (such as the abscopal effect of radiotherapy) [38] and promote the antitumor immunity to augment the therapeutic efficacy of IOT [39]. Currently, the most promising combination modalities for PD-1/PD-L1 inhibitors include other immune checkpoint inhibitors (namely CTLA-4 inhibitors), chemotherapy, and targeted therapies [40]. The rationales behind these combinations are multifactorial and include activating T cells by regulating different signaling pathways (the CTLA-4 checkpoint is critical for T cell priming and activation, whereas PD-1 blocks effector T cell responses in tissues), enhancing tumor antigen expression, exposing more new antigen mutations and higher mutation burdens, inducing PD-L1 expression, augmenting T cell infiltration around the metastatic sites, and producing a more favorable tumor microenvironment [1, 41]. Currently, a substantial number of trials have reported excellent outcomes of these combination modalities in several cancer types [21–30], which facilitated FDA approval of the nivolumab plus ipilimumab combination as first-line treatment for *BRAF* V600 wild-type unresectable or metastatic melanoma [42] and pembrolizumab plus pemetrexed and carboplatin as first-line treatment for metastatic NSCLC, irrespective of PD-L1 expression [28]. In the present study, pooled analysis of the 12 RCT also showed consistent benefit on tumor response, progression-free survival, and overall survival in IOCT-treated patients. The overall survival benefit of IOCT was identified across different combination modalities. These findings provide critical evidence for the clinical utility of IOCT in treating cancer, and we propose that trials with more combinational modalities in extensive tumor types be conducted.

Our study provides important evidence for broadening the scope of IOT application in even patients with low PD-L1 expression. This is of great clinical significance, as PD-1 and PD-L1 inhibitor first-line monotherapy is currently limited to patients with high PD-L1 expression, while most patients, such as those with metastatic NSCLC, have tumors with low or negative PD-L1 expression [43]. Numerous studies have examined the role of PD-L1 expression as a predictive biomarker of tumor response; nonetheless, its predictive value remains unclear due to the different cut-off values (e.g., 1%, 5%, 10%, and 50%) reported for the definition of PD-L1 positivity or negativity, which is further compounded by the possibility of interlaboratory variation [23–25, 43]. IOCT may help to overcome these issues through the immunomodulatory effect; inducing PD-L1 expression in tumor cells and exposing tumor antigens to immunocytes [1, 39, 41]. To validate the synergistic effect of immunotherapy and combinational therapies, we relied on a large dataset of 5388 cases, for all of whom the outcomes of a well-defined endpoint (overall survival) was reported. Through pooled analyses, we observed that although patients with high PD-L1 expression did better, those with low PD-L1 expression also benefit from the IOCT. We believe that the favorable overall survival achieved in the patients with low PD-L1 expression is mainly due to the immunomodulatory effect of the combinational therapies, which still rely on the biological function of the PD-1 or PD-L1 pathway and the complicated interaction between cancer cells and the immune system.

The AEs related to the PD-1/PD-L1 checkpoint inhibitors are generally considered well-tolerated and manageable, particularly when compared to the toxicity profile of other immunotherapy drugs such as CTLA-4 inhibitors and chemotherapy [44]. In IOCT, although the profile of AEs is similar to that observed in monotherapy and was generally reversible, the overall incidence and the incidence of high-grade AEs increased significantly. Therefore, we should be aware of the incidence and appropriate management of severe AEs caused by IOCT, especially the immune-related AEs such as immune-related myocarditis [45] and meningitis [46], when conducting a clinical trial or in conventional clinical use.

Our study has some notable limitations. First, there is moderate heterogeneity among the eligible studies (*P* = 0.054, *I*^2^ = 42.0%). However, the actual heterogeneity could be higher than the statistical heterogeneity. We believed that the heterogeneity is derived from the multiple intervention modalities (combinational therapies and PD-1/PD-L1 inhibitors) and various tumor types (NSCLC, melanoma, sarcoma, and others); therefore, although we performed subgroup analyses to mitigate that limitation, high heterogeneity should be noted when interpreting our results. Then, some of the eligible trials had a small number of participants and short follow-up duration, which may have resulted in fairly wide CIs of HRs for the treatment effects, thereby confounding our pooled HR results. Nonetheless, in sensitivity analyses restricted to large-scale trials (>400 participants) and trials with a long follow-up duration (>24 months), consistent survival advantages (large-scale trials: pooled HR: 0.66, *P* < 0.001; long follow-up duration trials: pooled HR: 0.68, *P* < 0.001; Figure 3) persisted. Next, this is a meta-analysis at trial level rather than individual level, the detailed information on patients that may impact the efficacy of immunotherapy, such as patient demographic (age and sex), was not available. Lastly, some trials had a crossover design, which could have weakened the treatment effect of IOCT. Nonetheless, we observed favorable results in this meta-analysis.

In conclusion, compared with PD-1/PD-L1 inhibitor monotherapy and conventional therapies, IOCT significantly prolonged overall survival in patients with advanced solid tumors regardless of cancer type and PD-L1 expression. However, we should note the increased incidence rate of high-grade AEs in IOCT. The present study findings could also aid clinicians in the clinical practice use of IOCT.

## Figures and Tables

**Table 1:** Characteristics of the RCT.

Study	Trial duration	Study type	Population	Treatment line	Experimental arm	Control arm	*N*	Follow-up (months)	Median OS (months)	High-grade AEs (no.) IOCT/non-IOCT
Antonia (2016)	2013-2015	Phase 1/2	SCLC	≥2	Nivolumab + ipilimumab^∗^	Nivolumab	213	12.0 vs. 8.67	6.0 vs. 4.4	10 vs. 13
Hodi (2016)	2013-2014	Phase 2	Melanoma	1	Nivolumab + ipilimumab	Ipilimumab	142	24.5	NR for both arms	51 vs. 9
Langer (2016)	2014-2016	Phase 2	NSCLC	1	Pembrolizumab + carboplatin + pemetrexed	Carboplatin + pemetrexed	123	10.6	NR for both arms	23 vs. 16
Wolchok (2017)	2013-2014	Phase 3	Melanoma	1	Nivolumab + ipilimumab	Ipilimumab; nivolumab	945	35.7 vs. 38 vs. 18.6	37.6 vs. NR vs. 19.9	184 vs. 86 vs. 67
D'Angelo (2018)	2015-2016	Phase 2	Sarcoma	≥2	Nivolumab + ipilimumab	Nivolumab	83	14.2	14.3 vs. 10.7	6 vs. 3
Gandhi (2018)	2016-2017	Phase 3	NSCLC	1	Chemotherapy + pembrolizumab	Chemotherapy + placebo	616	10.5	NR vs. 11.3	36 vs. 9
Long (2018)	2014-2017	Phase 2	Melanoma with brain metastases	≥1	Nivolumab + ipilimumab	Nivolumab	79	17	18.5 vs. NR	22 vs. 9
Socinski (2018)	2015-2016	Phase 3	NSCLL	1	Atezolizumab + bevacizumab + carboplatin + paclitaxel	Bevacizumab + carboplatin + paclitaxel	692	15.4	19.2 vs. 14.7	230 vs. 197
Horn (2018)	2016-2017	Phase 3	SCLC	1	Atezolizumab + carboplatin + Etoposide	Placebo + carboplatin + Etoposide	403	13.9	12.3 vs. 10.3	115 vs. 113
Motzer (2018)	2014-2016	Phase 3	RCC	1	Nivolumab + ipilimumab	Sunitinib	847	25.2	NR vs. 26	250/547 vs. 335/535^#^
Antonia (2018)	2014-2016	Phase 3	NSCLC	1	Durvalumab + chemoradiotherapy	Placebo + chemoradiotherapy	713	25.2	NR vs. 38.7	145 (30.5%) vs. 61 (26.1%)^¶^
Paz-Ares (2018)	2016-2016	Phase 3	NSCLC	1	Chemotherapy + pembrolizumab	Chemotherapy + placebo	559	7.8	15.9 vs. 11.3	194 vs. 191

Abbreviations: NSCLC: nonsmall cell lung cancer; SCLC: small cell lung cancer; RCC: renal cell carcinoma; OS: overall survival; NR: not reached; AE: adverse event; IOCT: immuno-oncology combination treatment. ^∗^Experimental arm included two cohorts: 1 mg/kg nivolumab plus 3 mg/kg ipilimumab (61 patients) and 3 mg/kg nivolumab plus 1 mg/kg ipilimumab (54 patients). We included the latter cohort in the present study. ^#^The high-grade AEs were reported among the intention-to-treat population. One patient in the experimental arm and three patients in the control arm were excluded from the safety analysis.

## Data Availability

All data used to support the findings of this study are included within the article.

## Supplementary Materials

Supplementary Materials PubMed search terms. Supplementary Table 1: risk of bias of the included trials. Supplementary Figure S1: forest plot of RR comparing overall response (A), and HR comparing progression-free survival (B) in patients who received IOCT vs. non-IOCT. RR: relative risk; HR: hazard ratio; IOCT: immuno-oncology combination treatment, CI: confidence interval. Supplementary Figure S2: Begg's funnel plot for publication bias test. Each circle represents a separate study for indicated association. Horizontal line, mean effect size. HR: hazard ratio.
