# Supplementary material for: The Efficacy and Safety of PD-1/PD-L1 Inhibitors in Combination with Conventional Therapies for Advanced Solid Tumors: A Meta-Analysis
Source: Biomed Res Int. 2020 May 6;2020:5059079. doi: 10.1155/2020/5059079 (PMC7225910; doi:10.1155/2020/5059079)
Supplement: Supplementary Materials — PubMed search terms. Supplementary Table 1: risk of bias of the included trials. Supplementary Figure S1: forest plot of RR comparing overall response (A), and HR comparing progression-free survival (B) in patients who received IOCT vs. non-IOCT. RR: relative risk; HR: hazard ratio; IOCT: immuno-oncology combination treatment, CI: confidence interval. Supplementary Figure S2: Begg's funnel plot for publication bias test. Each circle represents a separate study for indicated association. Horizontal line, mean effect size. HR: hazard ratio. [file 5059079.f1.doc]

**Supplementary Material**

**PubMed search terms:**

(“nivolumab”[Supplementary Concept] OR “Nivolumab”[tiab] OR“Opdivo”[tiab] OR "MDX-1106”[tiab] OR “ONO-4538”[tiab] OR “BMS-936558”[tiab] OR “NIVO”[tiab] OR “pembrolizumab”[Supplementary Concept] OR “pembrolizumab”[tiab] OR “lambrolizumab”[tiab] OR “keytruda”[tiab] OR “MK-3475”[tiab] OR “SCH  900475”[tiab] OR “avelumab”[Supplementary Concept] OR “Avelumab”[tiab] OR “MSB0010718C”[tiab] OR “MPDL3280A”[Supplementary Concept] OR “MPDL3280A”[tiab] OR “atezolizumab”[tiab] OR “Tecentriq”[tiab] OR “RG7446”[tiab] OR “RO5541267”[tiab] OR “Durvalumab”[tiab] OR “MEDI4736”[tiab] OR “MEDI-4736”[tiab] OR checkpoint inhibitor*[tiab] OR “PD-1”[tiab] OR “PDL1”[tiab]) AND (Clinical Trial, Phase III[ptyp] OR “phase 3 clinical trial”[tiab] OR “phase III clinical trial”[tiab] OR “phase 3 trial”[tiab] OR “phase III trial”[tiab] OR “phase 3 clinical study”[tiab] OR “phase III clinical study”[tiab] OR “phase 3 study”[tiab] OR “phase III study”[tiab] OR “phase 3 randomized trial”[tiab] OR “phase III randomized trial”[tiab] OR Clinical Trial, Phase II[ptyp] OR “phase 2 clinical trial”[tiab] OR “phase II clinical trial”[tiab] OR “phase 2 trial”[tiab] OR “phase II trial”[tiab] OR “phase 2 clinical study”[tiab] OR “phase II clinical study”[tiab] OR “phase 2 randomized trial”[tiab] OR “phase II randomized  trial”[tiab] OR “phase 2 study”[tiab] OR “phase II study”[tiab] OR “phase 2/3 clinical trial”[tiab] OR “phase II/III clinical trial”[tiab] OR “phase 2/3 trial”[tiab] OR “phase II/III trial”[tiab] OR “phase 2/3 clinical study”[tiab] OR “phase II/ III clinical study”[tiab] OR “phase 2/3 study”[tiab] OR “phase II/III study”[tiab] OR “phase 2/3 randomized trial”[tiab] OR “phase II/III randomized trial”[tiab] OR Randomized  Controlled Trial[ptyp] OR “randomized controlled trial”[tiab] OR “RCT”[tiab])

**Supplementary Table 1. Risk of bias of the included trials.**

| **Study** | **Randomization** | **Allocation concealment** | **Blinding** | **Incomplete outcome data** | **Selective outcome reporting** | **Other sources of bias** |
| --- | --- | --- | --- | --- | --- | --- |
| CheckMate 032 | Low | Low | High | Low | Low | Low |
| CheckMate 069 | Low | Low | Low | Low | Low | Low |
| KEYHighTE-021 | Low | Low | High | Low | Low | Low |
| NCT02374242 | Low | Low | High | Low | Low | Low |
| Impower 150 | Unclear | Unclear | High | Low | High | Low |
| PACIFIC | Unclear | Unclear | Low | Low | Low | Low |
| NCT02500797 | Low | Low | High | Low | Low | Low |
| KEYHighTE-189 | Low | Low | Low | Low | Low | Low |
| Impower 133 | Unclear | Unclear | Low | Low | Low | Low |
| KEYHighTE-407 | Low | Low | Low | Low | Low | Low |
| CheckMate 214 | Low | Low | High | Low | Low | Low |
| CheckMate 067 | Unclear | Unclear | Low | Low | Low | Low |

**Supplementary Figure S1.** Forest plot of RR comparing overall response (A), and HR comparing progression-free survival (B) in patients who received IOCT vs. non-IOCT. Studies are listed on the left with respective number of patients of each treatment, treatment effect with 95% CI and weight are on the right. RR, relative risk; HR, hazard ratio; IOCT, immuno-oncology combination treatment, CI, confidence interval

**
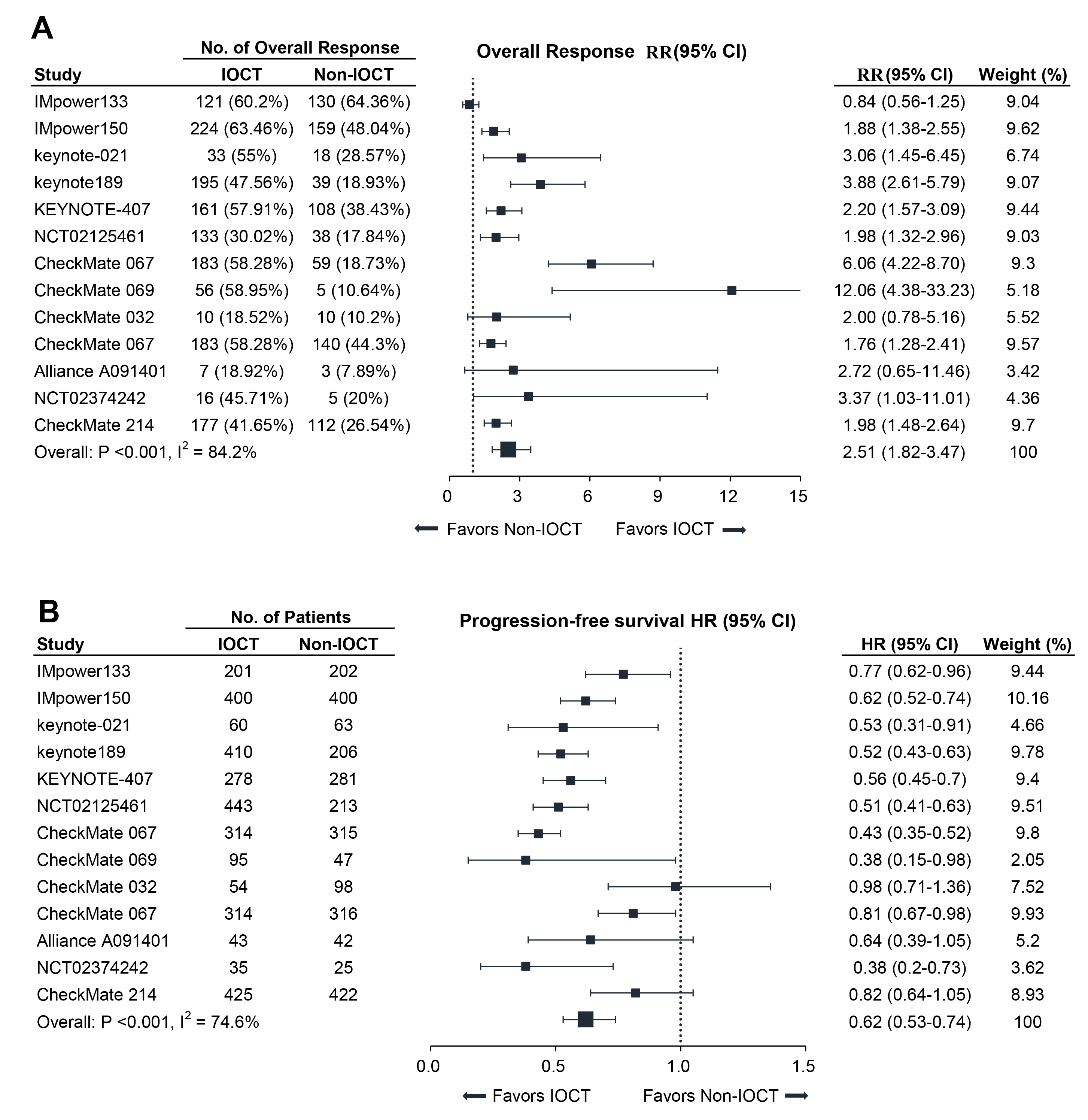
**

**Supplementary Figure S2.** Begg’s funnel plot for publication bias test. Each circle represents a separate study for indicated association. Horizontal line, mean effect size. HR, hazard ratio
